# A Nuanced Examination of Primate Capture and Consumption and Human Socio-Economic Well-Being in Kirindy Mitea National Park, Madagascar

**DOI:** 10.3390/ani13182914

**Published:** 2023-09-14

**Authors:** Domenic Romanello, Katharine E. T. Thompson, Cortni Borgerson, Jeanne Mathilde Randriamanetsy, Niaina Nirina Mahefa Andriamavosoloarisoa, Mamy Yves Andrianantenaina, Théofrico Alexander Razafindrahasy, Claire Surkis, Patricia C. Wright, Katheryn C. Twiss, Rebecca J. Lewis

**Affiliations:** 1 Department of Anthropology, University of Texas at Austin, 2201 Speedway, Stop C3200, Austin, TX 78712, USA; rjlewis@austin.utexas.edu; 2 Department of Anthropology, Stony Brook University, 101 Circle Rd, SBS Building S-501, Stony Brook, NY 11794, USAkatheryn.twiss@stonybrook.edu (K.C.T.); 3 Department of Anthropology, The Pennsylvania State University, 410 Carpenter Building, University Park, State College, PA 16802, USA; 4 The Climate School, Columbia University, Milstein Building, 177 Fort Washington Ave, New York, NY 10032, USA; 5 Department of Anthropology, Montclair State University, 1 Normal Ave, Montclair, NJ 07043, USA; borgersonc@mail.montclair.edu; 6 Madagascar Health and Environmental Research (MAHERY), Maroantsetra 512, Madagascar; 7 Zoology and Animal Biodiversity, University of Antananarivo, Antananarivo 101, Madagascar; 8 Tamàna Tour, Immeuble S, Ankorondrano, Alarobia, Antananarivo 101, Madagascar; 9 The Interdepartmental Doctoral Program in Anthropological Sciences (IDPAS), 101 Circle Rd, SBS Building S-501, Stony Brook, NY 11794, USA

**Keywords:** Africa, bushmeat, conservation, econometrics, hunting, income, lemur, poaching, poverty

## Abstract

**Simple Summary:**

Lemur hunting and consumption is widespread in Madagascar. Understanding what drives this phenomenon is key to conserving lemurs and protecting human livelihoods. Current research shows poverty to be a primary driver of lemur hunting and consumption, but no study has employed a composite poverty metric that includes health, education, and living standards. Our study employs such a metric, along with income, to investigate, for the first time, poverty as a driver of lemur hunting and consumption in and around Kirindy Mitea National Park, Madagascar. We document alarming levels of poverty and lemur hunting and consumption among the study community but find no relationship between the variables. Because nearly all households in the study community were impoverished, possible explanations include the following: (1) poverty is not a driver of lemur hunting and consumption in Kirindy Mitea National Park, or (2) a relationship could not be found due to the socio-economic similarity of households in our study community. We consider both interpretations in light of the current literature and offer recommendations for future research. If human and non-human primates are to continue to co-exist in and around Kirindy Mitea National Park, immediate investment in poverty eradication is required, and conservationists should test the efficacy of livestock interventions locally.

**Abstract:**

The futures of human and nonhuman primates are closely tied in protected areas. Understanding this interconnectedness is especially urgent in Madagascar, one of the world’s most impoverished biodiversity hotspots. Yet, no study has evaluated the relationship between poverty and lemur hunting and consumption using a composite poverty metric that includes health, education, and living standards. To address this gap, and to inform primate conservation practice and policy, we administered annual surveys to 81 households over six consecutive months (September 2018 to March 2019) in a village on the border of Kirindy Mitea National Park, Madagascar. We observed extreme deprivation scores across multiple dimensions of poverty and identified ninety-five percent of households as ‘impoverished’. Of these, three-quarters (77%) of households were identified as being in ‘severe poverty’. One-fifth (19%) of all households hunted lemurs and half (49%) of households consumed lemurs. While poverty eradication is an urgent need in communities around Kirindy Mitea National Park, our findings show no relationship between poverty and lemur hunting and consumption, perhaps due to the lack of variance in poverty. Our results highlight the need to investigate other contributory factors to lemur hunting and consumption locally. Because food insecurity is a known driver of lemur hunting and consumption among the study community, and because domestic meats can be preferred over protected species, we recommend testing the efficacy of livestock interventions near Kirindy Mitea National Park.

## 1. Introduction

Primate hunting is a global environmental and humanitarian problem [1]. Sixty-five percent of primate species are at risk of extinction (vulnerable—20%, endangered—28%, critically endangered—17%) [2], and hunting is a leading cause of primate population decline [3,4]. Reductions in primate populations not only harm ecological systems but also human sociocultural and socioeconomic systems [5]. Because of extreme poverty in many primate-range regions, primate hunting and consumption are useful coping strategies that provide essential income [4,6], calories, macronutrients (e.g., protein, fat), and micronutrients (e.g., zinc, iron) [7]. But when the effects of these coping strategies are combined with habitat loss and human population growth, hunting may become unsustainable [8]. As primate populations decline, food security is diminished, and local livelihoods become more precarious [9].

The problem is especially pertinent in Madagascar, a biodiversity hotspot in environmental and economic crisis [10,11,12,13]. Poverty is high in Madagascar, with 80% of its citizens living on less than USD 2.15 per day [14]. Food insecurity, malnutrition, and child mortality are likewise widespread [14,15,16]. Human population growth is rapid, and, taken together, these factors add pressure to already degraded environments [17]. The forests people require for their food, shelter, fire, medicines, and cultural significance are dwindling [18]. Madagascar’s forest ecosystems suffer from rampant deforestation [19,20]. Forest loss and fragmentation further exacerbate the effects of, and can even directly increase, hunting pressure [1]. Although Madagascar has legislation in place to protect its wildlife, the funding and logistical capacity necessary to enforce these laws is often lacking [12,21]. Even when laws are well enforced, people may still defy or ignore laws, especially if the policies do not address the underlying motives for hunting behavior [21]. Reliance on wildlife consumption is common in Madagascar, and prohibitions are often socially problematic, difficult to enforce, or unethical, as these restrictions worsen the prevalence and severity of malnutrition [22].

The key to wildlife conservation in Madagascar is understanding why people hunt and consume lemurs. Answering this question is urgent because all lemur species are experiencing population decline and 96% are threatened with extinction [2]. Numerous studies show poverty, poor health, food insecurity, and child malnutrition drive lemur hunting and consumption [23,24,25,26,27,28,29]. These findings are consistent with the broader literature that identifies poverty as an underlying driver of illegal wildlife hunting and consumption [30]. However, the relationship between poverty and lemur hunting and consumption is not well understood [31]. This lack of understanding is due, in part, to the complexity of the condition of poverty, and the diverse ways in which the term has been defined, operationalized, and measured [31].

The measurement of poverty has been both unidimensional and multidimensional. Unidimensional measures identify poverty based on a single variable, such as household income or household assets [32]. The multidimensional approach uses a variety of indicators in the measurement of poverty [33]. These indicators include things such as child mortality, school attendance, and access to clean drinking water [34]. The multidimensional approach offers a more robust assessment of poverty [35]. In this study, we use both the unidimensional (income) and the multidimensional approach for the assessment of poverty.

Conservationists typically employ unidimensional measures of poverty [36]. In a systematic review of the links between wildlife conservation and poverty, nearly three-quarters (70%) of published studies that addressed poverty as part of conservation used income as the primary measure [36]. Only one study has employed a multidimensional poverty metric (Multidimensional Poverty Index; MPI) to investigate illegal wildlife consumption in Madagascar; however, it did not include all dimensions, using just the living standards dimension and omitting the health and education dimensions [27]. Our study includes all dimensions of the MPI, including health, education, and living standards, to investigate poverty as a driver of lemur hunting and consumption.

To understand the impact of poverty on lemur hunting and consumption, we test the relationship between two measures of poverty [income and Multidimensional Poverty Index (MPI)]. First, we hypothesized a correlation between income and the MPI, and predicted that households with higher yearly per capita incomes would have lower MPI deprivation scores [35]. Second, we hypothesized that income and the MPI predict lemur hunting and consumption [25,27]. We expected that households with (a) higher yearly per capita incomes and (b) lower MPI deprivation scores are less likely to hunt and consume lemurs. We also predicted that (c) ‘impoverished’ households are less likely to hunt and consume lemurs than ‘severely impoverished’ households. Third, for the households that do hunt and consume lemurs, we hypothesized that income and the MPI predict the quantity of lemurs hunted and consumed [25,27]. We expected that households with (a) higher yearly per capita incomes and (b) lower MPI deprivation scores hunt and consume fewer lemurs. We also predicted that (c) ‘impoverished’ households hunt and consume fewer lemurs than ‘severely impoverished’ households.

## 2. Materials and Methods

### 2.1. Study Site

**Figure 1:**
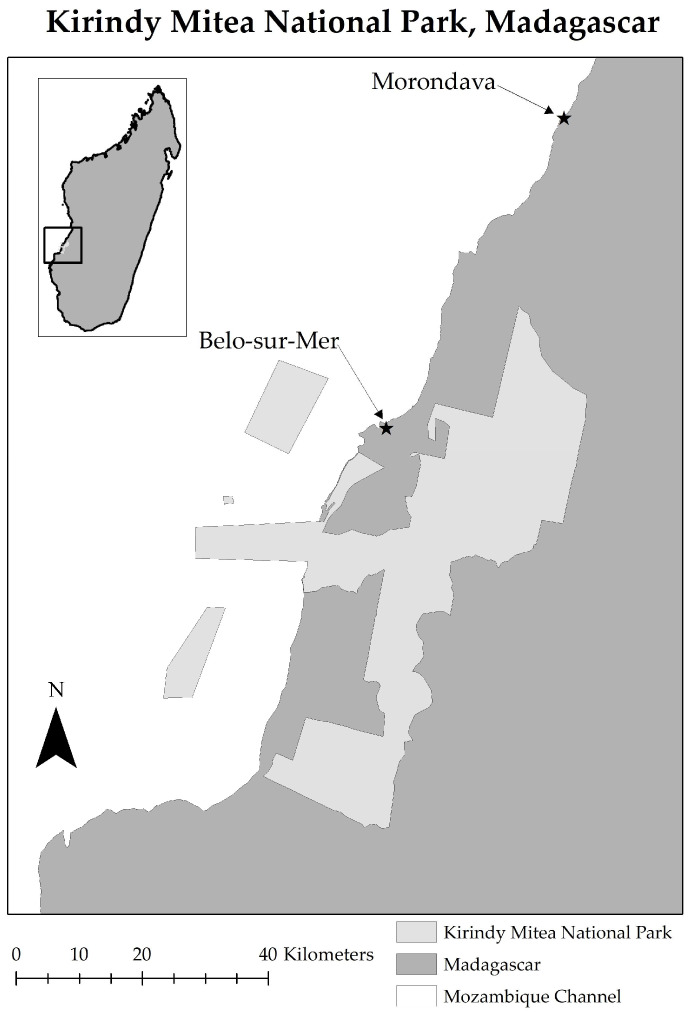
Map of Kirindy Mitea National Park, Madagascar, and the neighboring town of Belo-sur-Mer and city of Morondava.

Our study focuses on Kirindy Mitea National Park (Figure 1), Madagascar, a large (~140,000 ha), strictly protected area and UNESCO biosphere reserve with a high diversity of fauna (known species richness: birds—132, reptiles—31, bats—13, lemurs—8, amphibians—6, tenrec—5, carnivore—3) [37]. The protected area was established as a national park (IUCN category II) in 1997 to protect a critically important ecological transition zone between the dry deciduous forests of northwestern Madagascar, and the subarid spiny thickets of the southwest [38]. Kirindy Mitea National Park contains some of the largest tracts of dry forest remaining in Madagascar [39], but many threats to the protected area’s biodiversity exist: forest clearing, selective cutting, fire, the hunting of protected species, invasive species, and the collection of secondary products [37].

### 2.2. Study Population

We studied one subsistence community neighboring Kirindy Mitea National Park, in the southern Menabe region of Madagascar. Unlike other nations in Africa and Oceania, Madagascar is characterized by cultural unity and a shared mother tongue: Malagasy [40,41]. Regardless of dialectical differences or tribal identities, Malagasy people identify as Malagasy and share beliefs and practices associated with ancestors, divination, sacrifice, and spirits [41]. At our study site, the predominant cultural identities are mixed, with most inhabitants self-identifying as Sakalava-Vezo and/or Sakalava-Masikoro [29]. These cultural identities are not necessarily ethnicities, as cultural identity in Madagascar is often based upon kinship as well as a person’s occupation and place of residence [42]. The Sakalava-Vezo identify as fishermen, and the Sakalava-Masikoro primarily identify as farmers and herders [43]. Nevertheless, income modalities at the study site are complex. Households engage in agropastoral ventures but also depend on resources from the forest (i.e., timber, fruit, medicinal plants, and forest animals) and marine animals for both sustenance and sale. Opportunities for wage labor are limited beyond the seasonal harvest of salt, and minimal long-term employment exists in other extractive industries or ecotourism. Severe poverty, acute food insecurity, poor dietary diversity, and child malnutrition characterize the study community [29]. Wildlife hunting and consumption exceed levels documented in other conservation areas across Madagascar, and unlike other conservation areas in Madagascar, the most commonly hunted forest animals in Kirindy Mitea National Park are lemurs [29]. The hunting and consumption of lemurs in the park is driven, in part, by food insecurity [29]. Because households that consume lemurs report higher levels of food insecurity, lemur consumption is likely not a sufficient coping strategy [29]. While lemur consumption fails to alleviate food insecurity, lemurs still serve an important role locally, as a source of income and nutrients [29]. However, lemur hunting and consumption is likely less sustainable than the hunting of other species, such as bushpigs and tenrecs, due to the substantially lower annual reproductive rate of lemurs [29]. The study community is infrastructurally disconnected from nearby communities with commercial ties (such as Belo-sur-Mer) and larger urban areas (such as Morondava) due to the lack of paved roads and bridges. Many community members travel by boat, and travel by foot becomes more challenging in the rainy season. The infrastructural isolation of this community creates obstacles for the purchase of food, medicine, and other essential goods. No utility-grid electricity or running water is present at this site.

### 2.3. Ethical Note

The Stony Brook University Internal Review Board reviewed and approved all research methods (IRB #1183799-4). Before conducting this work, we acquired the requisite permits and approval from the Ministry of Environment and Forests and Madagascar National Parks at the national, regional, sub-regional, and local levels with the facilitation of Madagascar Institute for the Conservation of Tropical Environments (Permit #171/18/MEEF/SG/DGF/DSAP/SCB.Re). At the beginning of the study, we created a community map of the settlement that enumerated each household. We visited each household to enroll participants in the study. First, we read potential participants the informed consent documentation in Malagasy (with the help of Malagasy translators who had undergone ethics training). Exclusion criteria for this study included anyone who was imprisoned or who self-identified as suffering from (a) a severe mental or physical disability/illness (including Alzheimer’s, dementia, etc.), (b) a severe developmental disability, (c) a severe psychiatric illness, suffering from any alcohol or drug dependencies, or (d) a recent traumatic event (e.g., death in the family, domestic abuse). For children between 5 and 17 years of age, a parent provided informed, verbal consent in addition to the child’s assent. We did not obtain documented written consent for any of the participants due to low literacy and because signatures would have been inherently incriminating given the sensitive subject matter of the surveys. We informed all participants that they could withdraw at any point during the study, end an interview early, or skip any interview questions they did not feel comfortable answering. To ensure anonymity, we replaced the names of all individuals’ household and individual codes for data analysis and publication purposes (following Thompson et al., 2023) [29].

### 2.4. Data Collection Procedure

We collected data using annual household- and individual-level surveys over six consecutive months (September 2018 until March 2019), in the local dialect of Sakalava Malagasy. A full description of the design and implementation of these structured survey methods can be found in Thompson et al. (2023) [29]. For individual-level surveys (the data from which were aggregated to the household level for these analyses), we interviewed all adults and children over five years of age in each household. For household-level interviews, we asked the participating head-of-household to answer the questions on behalf of themselves and all other household members. Household-level interviews were conducted with one adult member (the self-identified male or female head of the household) from each of the 81 households (representing 339 individuals total). Each conversation took place inside or near each individual’s home (we deferred to the individual’s comfort level in order to protect their privacy) and lasted 30 to 90 min. During these interviews, we read the participant questions from an iPad and documented their responses using the Feed2Go survey app. If participants were not available, then we attempted to contact them on the following day; we visited all individuals over the course of the study.

### 2.5. Measures

#### 2.5.1. Lemur Hunting and Consumption

We measured lemur hunting and consumption during the prior year (the 12-month period preceding the date of interview). A ‘hunter’ is anyone who reported capturing at least one lemur in the prior year and a ‘consumer’ is anyone who reported eating any amount of lemur during the prior year. We used focus groups to confirm the local names of all lemur species at the beginning of the study, using laminated photo cards. Annual recalls for wildlife capture and consumption have been shown to produce reliable information in Madagascar [44]; nevertheless, participants may have underreported information given the conservation status of specific species. Therefore, we consider our results to represent minimum estimates of lemur utilization in Kirindy Mitea National Park.

#### 2.5.2. Income

We measured household income through summing the total amount of money earned from sales and wage labor by all household members during the prior year. Each source of income was quantified through asking participants the unit price, and number of units sold, of 23 commonly sold items (e.g., rice, timber, salt). We asked participants if they or anyone from the household earned a salary, and then asked how much they were paid per unit of time worked, the estimated number of units worked during the prior year, and whether it was related to national parks work or ecotourism.

#### 2.5.3. Poverty

We measured household poverty using the Multidimensional Poverty Index (MPI) [34]. The MPI measures three broad dimensions of poverty: health, education, and living standards. The health dimension consists of two indicators, nutrition (measured according to body mass index) and child mortality, while the education dimension consists of years of schooling and school attendance. The living standards dimension has six indicators: cooking fuel, sanitation, drinking water, electricity, housing, and assets. A household’s performance on each indicator is scored using a value of 1 or 0 (e.g., the household has access to clean drinking water—0; the household does not have access to clean drinking water—1). The value assigned to the indicator (0 or 1) is then multiplied by the indicator’s assigned weight (e.g., 1 × 1 ÷ 18; note: 1 ÷ 18 is the assigned weight for the drinking water indicator). The resulting values for each indicator are summed, yielding an MPI deprivation score, which ranges from 0 to 1. For households where nutritional (body mass index) data were not available, the child mortality indicator beared the full weight of the health dimension for calculation of the deprivation score. MPI deprivation scores are divided into three categories: ‘vulnerable to poverty’ (>0.20), ‘impoverished’ (>0.33), and ‘severely impoverished’ (>0.50). The MPI includes three indicators that are not applicable to all households: nutrition (body mass index), school attendance, and child mortality. In such cases, households without the relevant population are scored as non-deprived in the relevant indicators [35]. Deprivation cutoffs for all indicators are standard [34] except for child mortality. Due to data constraints, the deprivation cutoff for the child mortality indicator is as follows: a household is deprived if any mother has given birth to a child that has passed before their 18th birthday.

### 2.6. Data Analysis

We used R Statistical Software v.4.2.2. to explain the relationship between poverty and lemur hunting and consumption [45]. First, we evaluated the relationship between yearly per capita incomes and MPI deprivation scores at the household-level using Spearman’s rank correlation. Then, we tested yearly per capita incomes and MPI deprivation scores as drivers of household lemur hunting and consumption using a binomial generalized linear model (GLM). We then partitioned households experiencing poverty into two groups, ‘impoverished’ and ‘severely impoverished,’ and used a binomial GLM to further understand the effect of poverty on lemur hunting and consumption. Then, we tested yearly per capita income and MPI deprivation scores as drivers of variation in the number of lemurs hunted and consumed by households that did hunt and consume lemurs. To do this, zeros were excluded in the lemur hunting and consumption datasets, and the data were tested for overdispersion using a Poisson generalized linear model (GLM), which yielded overdispersion parameter values of θ > 2. We then used a quasi-Poisson GLM, which also yielded overdispersion parameter values of θ > 2. Ultimately, we used a negative binomial GLM because it yielded overdispersion parameter values of θ < 2. To further evaluate the drivers of variation in the number of lemurs hunted and consumed, we partioned households experiencing poverty into two groups, ‘impoverished’ and ‘severely impoverished,’ and compared between them using a nonparametric Kruskal–Wallis one-way analysis of variance on ranks.

## 3. Results

The study community was 44% (148) female and 56% (191) male. Ages ranged from 0 to 96 years with a mean ± SD of 24 ± 18 years.

### 3.1. Lemur Hunting and Consumption

One-fifth of households reported (19%) hunting lemurs, with a mean ± SD of 10 ± 9 lemurs hunted by each of these households during the previous year. Half of households (49%) consumed lemurs, with a mean ± SD of 9 ± 9 lemurs consumed by each of these households in the previous year (Table 1).

### 3.2. Income

Per capita household yearly income ranged from USD 1.66 to USD 1176.45 (conversion: MGA 1 = USD 0.00023) with a mean ± SD of USD 132.90 ± USD 196.53, and a median of USD 51.24. All households except one lived below the international poverty line of USD 2.15 a day and were experiencing ‘extreme income poverty’.

### 3.3. Poverty

Deprivation scores ranged from 0.22 to 1.00, with a mean ± SD of 0.59 ± 0.18. Nearly all households (95%) had MPI deprivation scores identifying them as ‘impoverished’ (>0.33), and three-quarters of ‘impoverished’ households (77%) were ‘severely impoverished’ (>0.50). Despite one-twentieth of households (5%) not being identified as ‘impoverished,’ they all were ‘vulnerable to poverty’ (deprivation score > 0.20).

#### 3.3.1. MPI Health Dimension

The MPI health dimension includes two indicators: nutrition (body mass index) and child mortality. Of the 49 households for which body mass index data were available, more than four-fifths (83.7%) were deprived in the nutrition indicator, meaning they had one or more malnourished individuals based on body mass index. One-quarter (23.5%) of households were deprived according to the child mortality indicator, meaning a mother in the household gave birth to a child that passed before turning 18.

#### 3.3.2. MPI Education Dimension

The second MPI dimension is education, which is also split into two indicators: years of schooling and school attendance. More than four-fifths (85.2%) of households were deprived according to the years of schooling indicator because no household member over 10 years of age completed six or more years of schooling. More than one-tenth (13.6%) of households were deprived based on the school attendance indicator, meaning one or more household members between the age of 6–14 years were not attending school.

#### 3.3.3. MPI Living Standards Dimension

The third MPI dimension is living standards, which is split into six indicators: cooking fuel, sanitation, drinking water, electricity, housing, and assets. All households were deprived according to the cooking fuel, sanitation, and drinking water indicators because all households cooked with wood or charcoal, no household had access to its own flush toilet or latrine, and water was acquired from an unprotected well with known contaminants. Three-quarters (76.5%) of households were deprived based on the electricity indicator because they did not have electricty in their home. Nine-tenths (88.9%) of households were deprived according to the housing indicator because their homes were made of rudimentary materials. Four-tenths (42%) of households were deprived in the assets dimension because they did not own one or more of the following items: radio, television, telephone, computer, animal cart, bicycle, motorbike, refrigerator, car, or truck.

### 3.4. The Relationship between Income and Poverty

**Figure 2:**
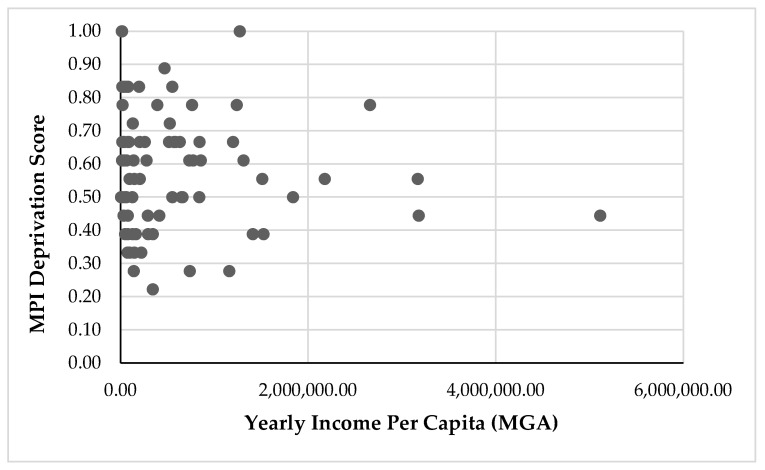
Scatterplot of Multidimensional Poverty Index deprivation scores and yearly income per capita in one community bordering Kirindy Mitea National Park (2018–2019).

Income and MPI deprivation scores were not significantly related (Figure 2; Spearman’s rank correlation test, r_s_ = −0.095, *p* = 0.399). Income did not significantly correlate with the health dimension (Spearman’s rank correlation test, r_s_ = 0.001, *p* = 0.998) or the education dimension (Spearman’s rank correlation test, r_s_ = −0.064, *p* = 0.568) but was weakly negatively correlated with the living standards dimension of the MPI (Spearman’s rank correlation test, r_s_ = −0.293, *p* = 0.008).

### 3.5. Poverty as a Driver of Lemur Hunting and Consumption

Poverty and lemur hunting and consumption were not significantly related. Higher yearly per capita incomes tended to decrease the probability that a household hunted lemurs, but the relationship was not significant (Table A1; binomial GLM, *z* = −1.678, *p* = 0.093). Income and lemur consumption were not significantly related (binomial GLM, *z* = −1.372, *p* = 0.170). Additionally, we found no significant relationship between MPI deprivation scores and lemur hunting (binomial GLM, *z* = −0.333, *p* = 0.739) and consumption (binomial GLM, *z* = −0.716, *p* = 0.474). Lemur hunting (binomial GLM, *z* = −0.094, *p* = 0.925) and consumption (binomial GLM, *z* = −0.063, *p* = 0.949) did not significantly differ between ‘impoverished’ and ‘severely impoverished’ households.

### 3.6. Poverty as a Driver of Variation in the Number of Lemurs Hunted and Consumed

Poverty was not significantly related to the number of lemurs hunted and consumed by households that hunted and consumed lemurs. No significant relationship was found between yearly per capita income and the number of lemurs hunted (negative binomial GLM, *z* = 0.783, *p* = 0.434) and consumed (negative binomial GLM, *z* = 0.418, *p* = 0.676). Additionally, MPI deprivation scores were not significantly related to the number of lemurs hunted (negative binomial GLM, *z* = 1.090, *p* = 0.276) and consumed (negative binomial GLM, *z* = −1.126, *p* = 0.260). Lemur hunting (Kruskal–Wallis rank sum test, x^2^ = 2.8017, df = 2, *p* = 0.246) and consumption (Kruskal–Wallis rank sum test, x^2^ = 0.173, df = 2, *p* = 0.917) did not significantly differ between ‘impoverished’ and ‘severely impoverished’ households.

## 4. Discussion

Our study evaluates the effect of poverty on lemur hunting and consumption in a national park in western Madagascar. Consistent with expectations, we observed high rates of poverty and lemur hunting and consumption. Contrary to expectations, we observed a lack of relationship between income and Multidimensional Poverty Index (MPI) deprivation scores and found that neither drove lemur hunting and consumption nor the variation in the number of lemurs hunted and consumed. Consequently, our results indicate that either (1) the conservationist assumption that poverty drives illegal wildlife hunting and consumption is not correct, or (2) poverty is a driver, but people living near Kirindy Mitea National Park, Madagascar, are so ubiquitously and severely impoverished that a relationship cannot be detected.

### 4.1. Rates of Poverty and Lemur Hunting and Consumption

In Kirindy Mitea National Park, Madagascar, rates of lemur hunting and consumption are high and exceed levels documented in other conservation areas in Madagascar (Table 1). At the same time, local incomes are extremely low. The proportion of households enduring severe income poverty (<USD 2.15 per capita per day) far exceeds the national average [14], with adverse consequences for human well-being and environmental resilience. Rates of multidimensional poverty are similarly elevated near Kirindy Mitea National Park. More than three-quarters (77%) of households were ‘severely impoverished’, surpassing national (45.5%), urban (20.8%), and rural (52.9%) estimates of severe multidimensional poverty [48]. The mean MPI deprivation score for our study population was also elevated (0.59) and exceeded national (0.38) and regional averages (0.47) [48].

### 4.2. The Relationship between Income and Poverty

Globally, and in Madagascar, the proportion of people that are multidimensionally poor correlates positively with the proportion of people that are unidimensionally poor [35]. Consistent with our hypothesis, the proportion of multidimensionally impoverished households (95%) was comparable to the proportion of unidimensionally impoverished households (99%). However, contrary to our hypothesis, we observed no correlation between income and MPI deprivation scores. We found a weak relationship between income and living standards among the study population, but household income had no relationship to overall MPI deprivation scores because we observed no relationship between household income and the health and education dimensions of poverty. Our findings bolster claims that income fails to capture the full scope of poverty people endure, highlighting the limitations of conclusions drawn from studies that employ income-based poverty metrics exclusively [31].

Poverty is more accurately measured multidimensionally because unidimensional measures, such as income, fail to capture the breadth with which poor people define their own poverty [34,49]. In communities neighboring Kirindy Mitea National Park, incomes are not only low; they also tend to be highly irregular and volatile—dependent upon the seasonal availability of things such as crop harvests and wage labor. Household incomes vary from day to day, season to season, and year to year. Our results suggest that these fluctuations, called income seasonality [50,51,52], may decrease the utility of income as a metric for household living standards. For example, a household could acquire solar electricity, or one of the assets of the MPI metric (e.g., phone) following the sale of a successful harvest. However, if that same household fails to generate substantial income throughout the rest of the year, it may be deemed unidimensionally poorer than a higher-income household without electricity. Our findings also show income to be a poor proxy for the health dimension of poverty in uniformly impoverished communities. For example, a high-income household may be unable to enjoy their relative wealth if they are unhealthy, and their slight income advantage does not afford them the necessary care [53]. Therefore, they may consider themselves poorer than lower income households. Furthermore, development efforts aimed towards health may increase life expectancy, or reduce child mortality rates, but may not affect household income [53]. Income is certainly an important contributory factor to health and longevity, but it is not the only contributory factor [54]. In impoverished communities with low income variability, small differences in income may not yield meaningful improvements to healthcare access. The same is likely true for the education dimension of poverty. For example, income may be a less important to children’s school attendance than other contributory factors (e.g., perceived importance of schooling, proximity to school) when income variance is low [55]. Conservationists examining the effect of poverty on protected areas should therefore measure poverty multidimensionally, especially in poor regions with low income diversity.

The lack of relationship between income and multidimensional poverty may also be due to the semi-subsistence livelihoods of our study community. Around Kirindy Mitea National Park, the extent to which households participate in the cash economy is variable. People near the park hunt, fish, and grow crops for subsistence. Rather than selling their goods to generate an income, people will often use their harvests directly or trade their harvests to acquire goods and services. Using income to measure poverty in semi-subsistence communities is sub-optimal because a household’s ability to improve its health, education, and living standards may not be dependent upon income but rather the goods a household harvests, uses, and trades [56]. Because people living in and around the world’s protected areas often have subsistence or semi-subsistence livelihoods [57], income alone is a poor metric of poverty to employ for conservation objectives.

### 4.3. Poverty as a Driver of Lemur Hunting and Consumption

Studies of illegal wildlife hunting and consumption identify poverty as a primary driver [58]. These studies often use income to measure poverty [59,60,61], but the MPI has also been shown to correlate with a perceived dependence on wild meats and an opposition to wild meat bans [62]. Our findings are divergent from the results of these studies and contrary to our hypotheses. We observed that income and the MPI were related to neither lemur hunting and consumption, nor the variation in the number of lemurs hunted and consumed. We consider the likelihood that poverty actually is a driver of lemur hunting and consumption, but that a relationship could not be detected due to low variance across income and MPI scores. Then, we discuss the implications of a true lack of relationship between poverty and lemur hunting and consumption.

All households in our study community were ‘vulnerable to poverty’, ‘impoverished’, or ‘severely impoverished’ based on MPI deprivation scores, and 99% of households were enduring severe income poverty. Thus, variance across annual household incomes and MPI deprivation scores was low [income range, USD 1.66 to USD 1176.45; income mean, USD 132.90; income standard deviation, ±USD 196.53; income median, USD 51.24 (conversion: MGA 1 = USD 0.00023); MPI range, 0.22 to 1.00; MPI mean, 0.59; MPI standard deviation, ±0.18] compared to national and international income distributions. Relationships between poverty and illegal wildlife hunting and consumption may be more easily detected in economically diverse communities (e.g., [59,60,63,64,65]). Equally, detecting a relationship between poverty and illegal wildlife hunting and consumption may be more challenging in homogenously impoverished communities and, therefore, around Malagasy protected areas in general [66]. No studies of illegal wildlife hunting and consumption in Madagascar have identified a relationship between income and lemur hunting and consumption [24,25,47]. Only one study links poverty and lemur hunting, and shows that in a small community of 36 households, lemur hunters were (1) sick more days in the prior month, (2) had lower quality homes, and (3) had children with lower body mass indices [25]. Likewise, a single study links poverty and lemur consumption, and shows that households with higher MPI deprivation scores (limited to the living standards dimension of the MPI) were more likely to consume lemurs [27]. However, this study found no relationship between poverty and lemur consumption in the Menabe region of Madagascar, where Kirindy Mitea National Park is located [27]. Based on the findings of our study, the absence of multidimensional poverty measures in the study of lemur hunting and consumption, and the existence of contradictory evidence on the relationship between poverty and lemur hunting and consumption, we consider the possibility that poverty is truly unrelated to lemur hunting and consumption.

One possibility is that livestock ownership is a more relevant driver of lemur hunting and consumption than poverty because households with access to domestic meats hunt and consume less wildlife [67,68]. Globally, the economic importance of illegal wildlife hunting and consumption is highest in poor communities with few domestic animals [69]. In Madagascar, livestock and fish are often preferred to protected species, such as lemurs [27], and access to these meats reduces food insecurity, a known driver of illegal wildlife hunting and consumption in Kirindy Mitea National Park and many other parts of Madagascar [25,29,46,70,71,72,73,74]. Our study community, characterized by diminutive differences in income and MPI deprivation scores, has substantial variation in livestock ownership that should be parsed to inform future conservation and development interventions. Research shows that livestock donations in exchange for not participating in illegal activities within protected areas are viewed as both fair and highly favorable by local community members [75].

Related to livestock is the issue of safety and security in and around Kirindy Mitea National Park. Livestock owners, and people in general, live in fear of dahalo. Dahalo are cattle rustlers notorious for heinous crimes including murder, rape, kidnapping, and theft [76,77]. Their relationship to both poverty and wildlife conservation is critical to consider given their ubiquity around Kirindy Mitea National Park. Dahalo exacerbate the effects of poverty locally because they steal livestock, money, and other personal property items, such as clothing and cooking materials (note that the actions of the dahalo themselves may be, in large part, poverty driven) [78]. Dahalo attacks lead to community displacement and overall declines in economic activity [78]. Households suffering the loss of livestock and other personal items may have an increased likelihood of hunting and consuming lemurs, firstly, as a coping strategy for the loss of meat and other resources, and secondly, as a result of proximity to the forest, because the park’s forest is used as refuge when villages are deemed unsafe. While the expansion of livestock ownership in communities around Kirindy Mitea National Park may yield positive results in the realms of food insecurity and wildlife conservation, the intervention is high-risk without improvements to local security. Community members near Kirindy Mitea National Park advocate for increased livestock availability, but many warn of the risks associated with cattle ownership in a region with dahalo. Based on conversations with local leaders, we recommend alternative protein support in the form of small livestock (e.g., goats, pigs, poultry, sheep) rather than cattle. Such an intervention has the potential to alleviate malnutrition, and the overexploitation of wildlife locally, but must be co-produced, workshopped, and subsequently assessed in partnership with the community [79]. Because the value of livestock is high, and households are able to supplement their diet with wild animals, it is conceivable that livestock will not be consumed or sold [80]. Therefore, we advocate for participatory engagement from community leaders and subsets of the population who may be affected differentially by the intervention, and we promote future qualitative research to build relationships and better understand local dynamics around wildlife hunting and consumption.

We also recommend consideration of sociocultural factors related to lemur harvests [81], as other places in Madagascar are likely to resemble our study community, i.e., socioculturally diverse and economically homogenous communities. In Madagascar, perceptions of wild meat hunters and consumers vary by species [82]. For example, the hunting and consumption of bushpigs is viewed with prestige, whereas the hunting and consumption of insectivorous bats is often viewed as shameful [82]. Cultural perceptions, especially those linked to the individually, ethnically, and regionally variable fady belief system likely also guide decisions around primate hunting and consumption [82]; this is documented in other publications on other regions of Madagascar [83,84,85], but is unfortunately not well documented or published in this study region. Future studies should explore and evaluate the relationship between belief systems and wild and domestic meat utilization in this area.

Finally, attention should be directed towards protected area governance and local perceptions/knowledge of institutions and legal frameworks related to protected area management. Globally, the economic importance of illegal wildlife hunting and consumption is high in impoverished communities under poor governance [69]. Corruption and the poor implementation of wildlife governance frameworks can negatively affect protected species generally [86] and reduce protected area efficacy in Madagascar [87]. Further, an individual’s decision to illegally harvest wildlife in a protected area can be influenced by their fear of recourse [88], but only if such fear outweighs the incentives to hunt. In the presence of corruption, if laws are not equitably enforced, and perceptions of penalties associated with law breaking vary substantially between individuals, some individuals can access wild meats without fear of recourse, while others cannot [69]. Governance, and local perceptions thereof, may affect the decision to illegally hunt or consume wildlife differently depending on one’s ability to influence such decision making. Further, given the severity of poverty in communities around Kirindy Mitea National Park endures, strict wildlife protections may be extraordinarily difficult for park agents who live in these communities to enforce [89], given that the incentives to hunt may outweigh the risk of such behaviors under current conditions. Rather than increasing enforcement, we advocate for immediate investment in poverty eradication, with a dual focus on interventions to increase livestock ownership while addressing dahalo-driven insecurity. Wildlife conservation in protected areas must be carried out, in part, to protect and sustain the livelihoods of local people.

## 5. Conclusions

Our findings have global ramifications for conservation research and policy. Unidimensional poverty measures, such as income, are frequently deployed in conservation research [36], and results inform critical policy decisions. Conservation policy may be misaligned with the core objectives of conservation if these findings are misinterpreted. Our results illustrate the inadequacy of income as a holistic poverty metric. In the future, conservationists should be cautious and clear about the ways in which they measure poverty, and multidimensional metrics should be deployed where possible.

Through using a holistic poverty metric, we observe an absence of a relationship between poverty and lemur hunting and consumption, with consequences for local policy. Because lemur harvests did not vary between ‘impoverished’ and ‘severely impoverished’ households, we do not advocate for poverty alleviation but rather investments in immediate poverty eradication and encourage further research into the efficacy of livestock interventions locally. The long-term resilience of Kirindy Mitea National Park, Madagascar, depends on the well-being of its primate inhabitants and human neighbors. The ubiquity and severity of poverty and lemur hunting and consumption in the park are alarming and have disastrous consequences for the region. Immediate intervention is necessary to alleviate wildlife utilization pressures and their underlying drivers.

## Figures and Tables

**Table 1:** Percentage of households that consumed lemurs in the prior year in one community bordering Kirindy Mitea National Park compared to other sites in Madagascar.

Study Sites	Percentage of Households That Consumed Lemurs
Kirindy Mitea National Park	49%
Masoala National Park [46]	19%
Ankarafantsika National Park [47]	11%
Kianjavato [24]	6%
Multi-site [27]	6%
Lac Alaotra [26]	3%

## Data Availability

Anonymized datasets are available from the corresponding authors upon reasonable request.

## Appendix A

**Table A1:** Income and poverty as potential drivers of lemur hunting and consumption in one community bordering Kirindy Mitea National Park (2018–2019).

Predictor Variable	Response Variable	Distribution	Coefficient Estimate	Standard Error	*z* Value	*p* Value
annual per capita income	lemur hunting (yes/no)	binomial	−1.451 × 10^−6^	8.648 × 10^−7^	−1.678	0.093
annual per capita income	lemur consumption (yes/no)	binomial	−4.211 × 10^−7^	3.069 × 10^−7^	−1.372	0.170
weighted household deprivation score (MPI)	lemur hunting (yes/no)	binomial	−5.417 × 10^−1^	1.628	−0.333	0.739
weighted household deprivation score (MPI)	lemur consumption (yes/no)	binomial	−9.133 × 10^−1^	1.272	−0.716	0.474
‘impoverished’ vs. ‘severely impoverished’	lemur hunting (yes/no)	binomial	−0.047	0.502	−0.094	0.925
‘impoverished’ vs. ‘severely impoverished’	lemur consumption (yes/no)	binomial	−0.025	0.395	−0.063	0.949
annual per capita income	number of lemurs hunted	negative binomial	5.117 × 10^−7^	6.536 × 10^−7^	0.783	0.434
annual per capita income	number of lemurs consumed	negative binomial	1.043 × 10^−7^	2.494 × 10^−7^	0.418	0.676
weighted household deprivation score (MPI)	number of lemurs hunted	negative binomial	1.528	1.402	1.090	0.276
weighted household deprivation score (MPI)	number of lemurs consumed	negative binomial	−9.845 × 10^−1^	8.743 × 10^−1^	−1.126	0.260
